# A new technique for assessing arterial pressure wave forms and central pressure with tissue Doppler

**DOI:** 10.1186/1476-7120-5-6

**Published:** 2007-01-31

**Authors:** Brian A Haluska, Leanne Jeffriess, Phillip M Mottram, Stephane G Carlier, Thomas H Marwick

**Affiliations:** 1 University of Queensland Department of Medicine, Princess Alexandra Hospital, Ipswich Rd, Brisbane Q4102, Australia; 2 The Cardiovascular Research Foundation, 55 East 59th St., 6th Fl., New York, NY 10022-1122, USA

## Abstract

**Background:**

Non-invasive assessment of arterial pressure wave forms using applanation tonometry of the radial or carotid arteries can be technically challenging and has not found wide clinical application. 2D imaging of the common carotid arteries is routinely used and we sought to determine whether arterial waveform measurements could be derived from tissue Doppler imaging (TDI) of the carotid artery.

**Methods:**

We studied 91 subjects (52 men, age 52 ± 14 years) with and without cardiovascular disease. Tonometry was performed on the carotid artery simultaneously with pulsed wave Doppler of the LVOT and acquired digitally. Longitudinal 2D images of the common carotid artery with and without TDI were also acquired digitally and both TDI and tonometry were calibrated using mean and diastolic cuff pressure and analysed off line.

**Results:**

Correlation between central pressure by TDI and tonometry was excellent for maximum pressure (r = 0.97, p < 0.0001). The mean differences between central pressures derived by TDI and tonometry were minimal (systolic 5.36 ± 5.5 mmHg; diastolic 1.2 ± 1.2 mmHg).

**Conclusion:**

Imaging of the common carotid artery motion with tissue Doppler may permit acquisition of a waveform analogous to that from tonometry. This method may simplify estimation of central arterial pressure and calculation of total arterial compliance.

## Background

Blood pressure has been shown to be a strong predictor of cardiovascular risk–increased systolic blood pressure (SBP) reflecting stiffening of the arterial walls and changes in vascular structure, and increased pulse pressure (PP) reflecting stiffening of conduit vessels [1,2]. Arterial stiffness may now be measured as a marker of arterial health [3], and in combination with cardiac output can be used to calculate total arterial compliance. This is usually performed using applanation tonometry of the radial artery and the use of a transfer function to estimate central pressure, but there are potential problems imposed by the transfer function [4-6] and direct acquisition of a central waveform might avoid them. However, acquisition of arterial waveforms obtained by applanation tonometry assumes that the artery is compressible [5,7], which may not be true for the carotid artery. Moreover, the adoption of this technique has been adversely impacted by lack of familiarity with tonometry and the need to obtain specialized equipment.

In contrast, carotid imaging is very familiar. Assessment of carotid intima-media thickness (IMT) is well established [8,9], has been used as a marker of atherosclerotic burden, and has prognostic value [10]. The use of the same imaging test to obtain arterial waveforms could enhance the anatomic evaluation of carotid IMT with information about arterial function. Doppler echocardiography, used traditionally to evaluate the velocity and direction of blood flow in the heart and vessels, can be used to evaluate low velocity, high amplitude signals which come from tissue by reduction of the wall filters and scale [11,12]. The use of color tissue Doppler imaging (TDI) permits rapid, simultaneous visualization of multiple structures in a single view. To date, the main cardiovascular application of this technique has been in myocardial tissue characterization [13]. In this study, we sought whether assessment of central pressure using displacement measured by TDI correlated with measures attained by applanation tonometry, and could be useful in assessing patients at risk for cardiovascular disease.

## Methods

### Patient selection

We studied 91 subjects (52 men, age 52 ± 14 years) with and without cardiovascular disease. Of these patients, 33 were normal controls (NL), 28 were patients with isolated systolic hypertension (HTN), 10 were normotensive patients with diabetes mellitus (DM), 10 were patients with systolic heart failure (CHF), and 10 were patients who had undergone renal transplantation (RT). Clinical data, blood pressure, and cardiovascular risk factors were gathered and all patients were then studied with simultaneous applanation tonometry and pulsed-wave Doppler and then had 2D ultrasound and tissue Doppler performed on their carotid artery.

### Applanation tonometry

**Figure 1:**
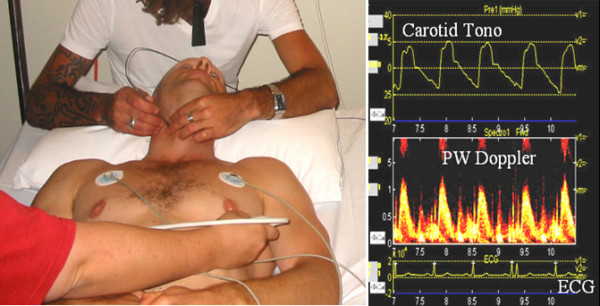
**Acquisition of carotid tonometry and pulsed Doppler**. Acquisition of simultaneous carotid tonometry and pulsed Doppler of the LVOT (left) and digitized raw data (right) showing carotid tonometry, reconstructed pulsed-wave Doppler and ECG.

Applanation tonometry was performed on the right carotid artery in all patients after they had been allowed to rest for 5–10 minutes. Tonometry uses a transcutaneously applied, micro-manometer-tipped probe which is placed against an arterial wall. Application of sufficient pressure to distort, or applanate the artery creates a signal which approximates instantaneous arterial pressure (Figure 1). This is then digitized and reconstructed on a personal computer. Blood pressure was measured using a standard sphygmomanometer on the right brachial artery, after the patient was allowed to rest 5–10 minutes. Calibration of the tonometric waveform was performed by assuming equivalence of mean [(2*DBP + SBP)/3] and diastolic brachial cuff pressure.

### Calculation of arterial compliance

Total arterial compliance (TAC) was derived using the pulse pressure method, using a combination of stroke volume (derived by echocardiography from the dimensions and pulsed-wave Doppler measurement of flow in the left ventricular outflow tract (LVOT)) and the pressure waveform obtained from applanation tonometry of the radial artery [14]. These waveforms were obtained simultaneously with the pulsed-wave Doppler, digitized (WaveBook 512, IOTech Inc., Cleveland, OH), and transferred to a laptop computer, where they were synchronized using the R wave of the electrocardiogram. Using specialized acquisition software, three sets of gated data (ECG, tonometry, Doppler) were acquired and stored for off-line analysis (figure 1). Depending on heart rate, this was usually 20–30 cardiac cycles per patient. Echocardiographic images and pulsed Doppler were acquired using a Philips HDI5000 ultrasound system (Philips Inc, Bothell, WA) with a 1.7 MHz harmonic imaging probe.

### Derivation of TAC

**Figure 2:**
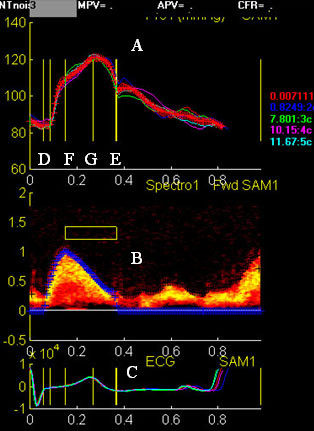
**SAM analysis window**. Analysis window for estimating total arterial compliance showing (A) averaged and calibrated carotid pressure waveform, (B) averaged reconstructed pulse-wave Doppler of the left ventricular outflow and (C) ECG. Cursors mark beginning (D) and end aortic ejection (E), peak aortic ejection (F) and peak pulse pressure (G).

For calculation of total arterial compliance, the binary files were processed and analyzed using a custom analysis program written in MatLab 4. Between five and ten cardiac cycles of tonometry and Doppler were chosen from the raw dataset based on data quality and averaged for analysis. With the use of cursors to identify the beginning and end of aortic ejection, peak ejection, and peak pulse pressure on both the reconstructed Doppler and central pressure waveforms, the analysis program determined mean values for pressure and flow and calculated a mean aortic pressure (figure 2). Based on the pressure and flow data derived from the central pressure waveform and the Doppler, the analysis program generated values for total arterial compliance, augmentation index, and other hemodynamic indices. We have previously reported the intra-observer variation (mean TAC 1.17 ± 0.02 ml/mmHg) and coefficient of variation of this method (1.7%) [15].

### Carotid TDI

**Figure 3:**
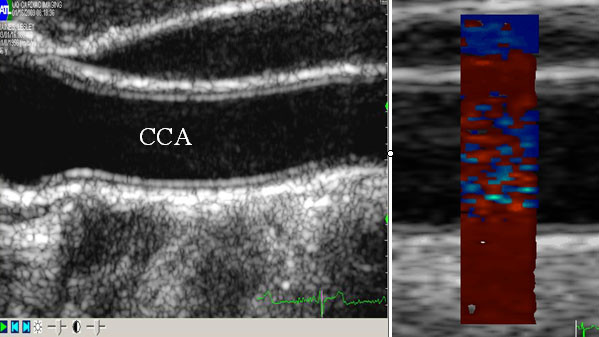
**Carotid 2D and TDI imaging**. Digitized longitudinal 2D scan of the common carotid artery (CCA) just prior to the bifurcation (right) and optimized ROI window with color tissue Doppler of the CCA.

The carotid arteries were scanned longitudinally in the anterior, lateral and posterior aspects 2–10 cm below the bifurcation, and digital cine loops are acquired for offline analysis. The image was then optimized for TDI using the smallest possible region of interest (ROI) box to achieve the highest frame rate, which was usually between 160–220 frames per second. The 2D and color Doppler settings were also customized for extraction of vessel displacement: for 2D imaging – dynamic range of 150 dB, the 2D option is set to penetration, persistence is set at low and frame rate is set at maximum; for the color tissue Doppler – the gain was set at 100%, with a PRF of >200 Hz. Care was taken not to include any discrete plaques in the tissue Doppler measurements. Loops of 3–5 cardiac cycles with TDI were then acquired digitally for offline analysis (figure 3).

### Derivation of vessel wall displacement

**Figure 4:**
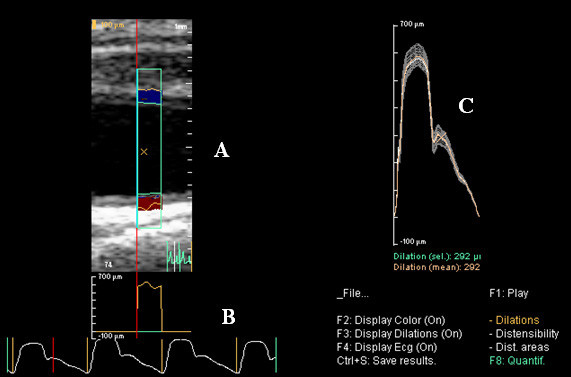
**Arterial wall motion (AWM) analysis window**. Arterial wall motion analysis window showing (A) the extracted arterial displacement over the cardiac cycle, (B) individual displacement curves over time for each cardiac cycle, and (C) the mean displacement for all of the cardiac cycles.

The TDI images were analyzed offline using custom-written software (AWM v1.05, Philips Medical Systems, Bothell, WA) which extracts the velocity information for the ROI area over the cardiac cycle, and with a processing algorithm, generates values for vessel wall displacement (in microns) over time [16]. These data were then saved and exported in numerical format for analysis (figure 4).

### Comparison of TDI displacement and tonometry

**Figure 5:**
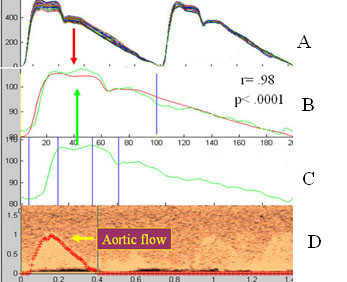
**SAMTDI analysis window**. Analysis window for comparison of tonometry and TDI showing (A) the extracted raw TDI displacement curves, (C) carotid tonometry arterial waveform, (B) calibrated TDI waveform (red) and carotid arterial waveform (green), and (D) reconstructed Doppler of aortic flow.

Both the extracted TDI displacement curves and the tonometric curves were then imported into a custom written MatLab program for analysis and once calibrated, a comparison was done between the waveforms (figure 5). Carotid displacement curves (in μm) derived from the TDI recordings were transformed into approximated pulse pressure curves (in mmHg) following the methodology described by Van Bortel et al based on the observation that the mean blood pressure is constant throughout the large artery tree [17], as well as the diastolic pressure [18]. The tonometric recordings were calibrated setting the average integrated curve equal to the mean blood pressure [derived from (2*DBP+SBP)/3] or if used, from an average automatic BP recording system. The minimum diastolic tonometric value was set equal to the diastolic BP. Similarly, a two-point calibration was used for the carotid distension curves, integrated with the mean set to the mean BP, and the minimum distension set to the diastolic BP.

### Statistical analysis

Pearson's correlation was used to determine the concordance between central pressure determined by TDI and by tonometry, paired t-tests were used to determine the mean differences between the two techniques and Bland-Altman analysis was used to determine the differences from the mean for central pressure between the two techniques.

## Results

### Patient characteristics

Table 1 shows clinical data, medications, blood pressure, flow and compliance for the whole patient group. Patients had an average of 1 ± 1 cardiovascular risk factors, and were on 1 ± 1 antihypertensive medications. However, blood pressure, cardiac output and TAC were all within the normal range.

**Table 1:** Patient characteristics

**n = 91**			
**Male Gender**	52 (57%)	**Age**	52 ± 14
**Smoking**	20(22%)	**SBP (mmHg)**	124 ± 22
**Hypertensive**	46 (50%)	**DBP (mmHg)**	76 ± 10
**DM**	15(16%)	**MAP (mmHg)**	92 ± 13
**Lipids**	30(33%)	**PP (mmHg)**	47 ± 18
**β Blocker**	20(22%)	**CO (L/min)**	4.6 ± 1.32
**CA**^++ ^**Blocker**	15(16%)	**TAC (ml/mmHg)**	1.48 ± .70
**ACE**	26(28%)	**# risk factors**	1 ± 1.2
**Statin**	29(32%)	**# medications**	1 ± 1.2

Clinical characteristics, medications, Systolic, diastolic, mean arterial and pulse blood pressure (SBP, DBP, MAP, PP), cardiac output (CO) and total arterial compliance (TAC).

### Comparison of central pressures obtained by tonometry and tissue Doppler imaging

**Figure 6:**
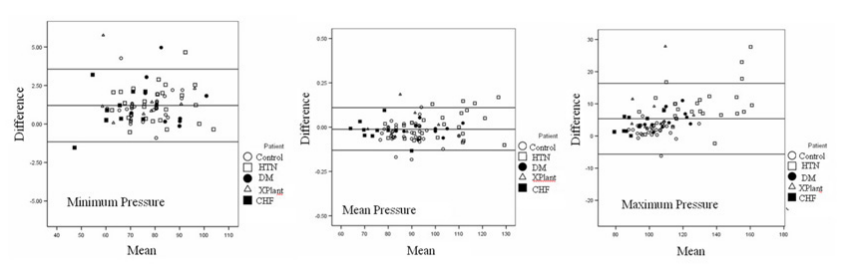
**Comparison of minimum, mean and maximum pressure**. Bland-Altman plots comparing mean pressure versus the difference in mean pressure for minimum, mean and maximum pressure.

Correlation between central pressure by TDI and tonometry was excellent for maximum pressure (r = 0.97, p < 0.0001). In paired t-tests the mean differences between central pressures derived by the two techniques was minimal, although there was a 5 mmHg difference between maximum pressure derived by TDI and tonometry (table 2). Bland-Altman analysis was then performed to determine the concordance between the two techniques. For diastolic pressure the differences were negligible (1.20 ± 1.2 mmHg) but this was expected since both sets of data were calibrated with diastolic cuff pressure (figure 6). For systolic pressure however the difference from the mean was greater (5.36 ± 5.5 mmHg) with the TDI underestimating maximum pressure (figure 6).

**Table 2:** Correlations and t-tests

	Tono	TDI	r	Difference	p
Maximum BP-mmHg	112 ± 20	107 ± 17	.97	5.36 ± 5.5	< .0001
Minimum BP-mmHg	77 ± 11	75 ± 10	.99	1.20 ± 1.2	< .0001
Mean BP-mmHg	92 ± 13	92 ± 13	1.00	-.01 ± .06	< .0001
Median BP-mmHg	89 ± 12	92 ± 13	.98	-2.96 ± 2.52	< .0001

Results of paired t-tests and Pearson's correlations between TDI and tonometry in the subgroups for maximum, minimum, mean and median blood pressure. Tono – tonometry; BP – blood pressure.

### Comparison of maximum pressure in subgroups

Because of the calibration with mean and diastolic pressure, only maximum pressure was compared in a subgroup analysis. The HTN group had the highest maximum pressure and the CHF patients had the lowest pressure, as would be expected. The controls, DM group and RT groups all had maximum pressure within a normal range. In paired t-tests comparing the TDI and tonometry the HTN group had the highest paired difference (9 ± 6 mmHg; p < 0.0001) and the controls had the smallest paired difference (2 ± 2 mmHg; p < 0.0001) (table 3).

**Table 3:** Correlations and t-tests

	Tono (mmHg)	TDI (mmHg)	Difference (mmHg)	p
Control (33)	103 ± 9	101 ± 9	2.08 ± 2.2	< .0001
HTN (28)	132 ± 22	122 ± 19	9.23 ± 6.3	< .0001
DM (10)	114 ± 10	109 ± 9	5.18 ± 3.0	< .0001
RT (10)	108 ± 12	100 ± 11	7.50 ± 7.7	< .01
CHF (10)	91 ± 10	87 ± 8	3.38 ± 2.6	< .003

Subgroup analysis and paired differences between carotid tonometry and tissue Doppler. Tono – tonometry

### Technical limitations of TDI approach

**Figure 7:**
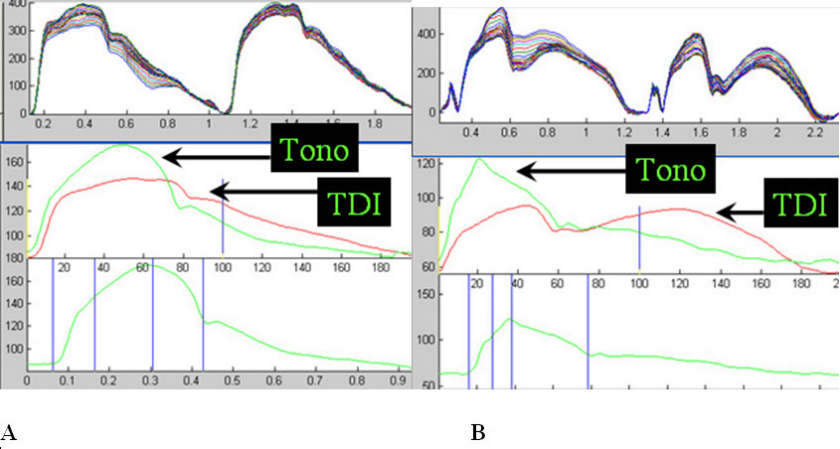
**Limitations – discordance**. Two examples of discordance in the comparison of central pressure obtained by tonometry and TDI. (A) Shows a patient in which the TDI velocities were aliased resulting in a gross underestimation of maximum (systolic) pressure. (B) Shows a patient with excessive movement of the carotid artery during acquisition of the TDI images resulting in artifact in the displacement waveform.

**Figure 8:**
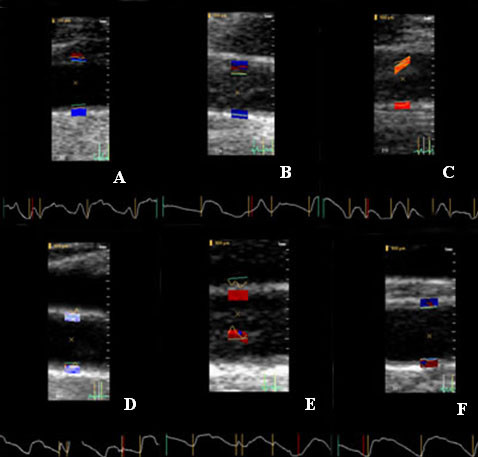
**Limitations – artifacts**. Artifacts with carotid tissue Doppler caused by technical issues: A – artifact from breathing which caused the artery to move in and out of the image plane; B – Excessive noise in the lumen which the analysis program picked up as tissue; C – Reverberation causing inaccurate detection of the vessel wall; D – Excessive movement of the artery during the cardiac cycle; E – Reverberation causing inaccurate detection of the vessel wall; F – Very little movement detected in the artery at peak pulse pressure and flow resulting in poor quality waveforms.

The TDI technique underestimated systolic pressure in about a third of cases resulting in a higher than acceptable difference in the Bland-Altman analysis. The causes of this appear to be related to inappropriate TDI settings and other technical problems, and therefore this may be avoided with experience and guidelines. Failure to use a sufficiently high pulse repetition frequency caused aliasing of the velocity signals and the resulting displacement waveforms and calibration by the technique described above produced a pressure waveform which does not reflect accurate maximum pressure when compared to the carotid tonometry in several cases (Figure 7). Excessive motion of the carotid artery may lead to failure of the edge detection mechanism. This excessive motion has affected the reproducibility of carotid tonometry in other studies and can be seen here as well (Figure 7) and may be controlled by a simple breath hold or transducer immobilization. Other causes of failure of the edge detection with TDI are reverberation artifacts, excessive noise in the vessel lumen and excessive vessel movement due to breathing or high mobility of the carotid artery (Figure 8). Once again these may be able to be corrected with breath holds, patient positioning, and careful attention to 2D and Doppler settings. Thus, with attention to technical aspects and patient preparation this technique is likely to be highly feasible.

## Discussion

The results of this study suggest that carotid TDI can be used to approximate central blood pressure and that the resulting waveform is analogous to that obtained with applanation tonometry. This would obviate the need for a transfer function and the waveform could be used to calculate total arterial compliance from central rather than peripheral pressure. The advantage of TDI imaging is that vascular imaging is available, feasible in a clinical setting, and can be incorporated into any cardiovascular imaging examination without extra equipment.

### Significance of arterial properties

Total arterial compliance is reduced with age, vascular disease and hypertension [19-21], and is linked with the sequellae of these disorders. Reduction of compliance leads to an increase in afterload on the heart, an increase in pulse pressure and in turn, left ventricular hypertrophy [22] and diastolic dysfunction. Increased pulse pressure is a determinant of cardiovascular risk and mortality [2,23-25]. Lower compliance also leads to lower diastolic pressure [26], resulting in a decreased coronary perfusion pressure [27]. Several studies have shown a correlation between compliance and the presence of significant CAD [28-30] as might well be expected due to common cardiovascular risk factors. Moreover, arterial compliance has been shown to be a contributor to the provocation of ischemia at stress testing [31], and an association has been documented between reduced exercise capacity and the presence of a lower ischemic threshold [32,33].

While brachial blood pressure has been shown to be a strong predictor of cardiovascular morbidity, mortality and outcome [2,22,34,35] the role of central pressure in clinical outcomes is increasingly recognized [35]. Independent of brachial blood pressure, central pulse pressure predicts left ventricular structure [36,37] and the extent of coronary artery disease. The CAFÉ substudy of the ASCOT trial showed that central pressure and pressure augmentation were significantly associated with clinical endpoints [35]. Despite insignificant differences between brachial pressures in the study groups over a four year follow-up period, the group with lower central pulse pressure showed a significant decrease in cardiovascular events. From the results of this study, assessment of central blood pressure appears to be paramount in the treatment of patients at risk for cardiovascular disease.

### Limitations of applanation tonometry

While applanation tonometry is considered the "gold standard" for use in assessing arterial stiffness it is not without limitations. The major limitation of pressure measurement with tonometry is the use of brachial cuff pressure to calibrate the signals. Past studies have shown poor inter-observer variability in taking brachial pressure and a poor correlation between reported and actual pressure [38,39]. Operator error and a learning curve with acquisition of tonometry also play a role in its usefulness as a clinical tool. And while several studies have shown the limitations of using transfer functions with radial tonometry {4, 5, 6, 7, 14}, the issues of performing carotid tonometry remain–lack of bony support underneath to achieve true applanation, obesity, respiratory changes and mobility of the artery

## Conclusion

The results of this study suggest that tissue Doppler may be useful in assessing central arterial pressure and in the estimation of total arterial compliance. Carotid imaging for IMT is familiar and feasible and has already been incorporated into many cardiovascular imaging laboratories. The use of the same imaging test to obtain arterial waveforms could enhance the evaluation of carotid IMT with information about arterial function as well. In addition, calibration of the arterial displacement waveforms obtained from TDI may simplify the estimation of total arterial compliance and alleviate the technical considerations of tonometry.

## Competing interests

The author(s) declare that they have no competing interests.

## Authors' contributions

BAH conceived the study design, gathered and analyzed the data, interpreted the results and drafted the manuscript

LJ assisted in gathering the tonometry and TDI data

PMM assisted in gathering the carotid tonometry and in recruiting patients for the study.

SGC developed the acquisition and analysis software for the tonometry and wrote the analysis program to compare the tonometry and TDI

THM was instrumental in study design and implementation, helped to interpret the results and edited the final manuscript
